# CAD/CAM and biomechanical simulations vs. standard technique for the design of braces in adolescent idiopathic scoliosis: first results

**DOI:** 10.1186/1748-7161-8-S1-O41

**Published:** 2013-06-03

**Authors:** F Desbiens-Blais, J Clin, S Parent, H Labelle, CE Aubin

**Affiliations:** 1 Ecole Polytechnique de Montreal, Montreal, Canada; 2 CHU Sainte-Justine, Montreal, Canada

## Background

Brace design is mostly done using empirical methods. Recent advances in CAD/CAM and computer biomechanical simulations now allow designing, optimizing and fabricating a novel generation of braces with expected biomechanical improvements [1].

## Aim

This study Aims at comparing the effectiveness of this new brace design technique to a standard technique (TLSO) system for the treatment of adolescent idiopathic scoliosis (AIS).

## Methods

So far, 4 AIS patients were recruited. Two braces were built for each patient: 1) a standard TLSO designed using the plaster-cast method (Std Brace), and 2) a brace designed and fabricated using a biomechanical 3D finite element model personalized to the patient’s geometry, and a CAD/CAM software (NewBrace). NewBrace was optimized using a computational brace simulator that allows virtual installation of the brace on the patient model, and prediction of its effect before fabrication. Several virtual braces were thus iteratively tested, and the one giving the best immediate correction was chosen for refinement by the orthotist, and fabricated using a computer-aided carver. Immediate brace effectiveness was assessed using radiographs in both braces.

## Results

For the first 4 patients, the Std Brace corrected the thoracic and lumbar Cobb angles by 51% and 45% respectively, while NewBrace corrected these angles by 41% and 48%. There was little effect of the sagittal curves, and both braces maintained the coronal balance. The patients were more comfortable in the NewBrace. The predictions of the brace simulator were found to be reliable.

## Conclusion

These first results showed the feasibility of a new technique, to design braces using a biomechanical simulation tool, and assess their effectiveness with respect to current design standards. The optimization process of the brace design is currently being improved. An extended study on more cases (10 to 15 patients should be included in the study before May) is under way to fully assess this new design paradigm.
